# Adipokine, endocrine, and fibrinolysis-related alterations across abdominal obesity and metabolic syndrome phenotypes in young adults

**DOI:** 10.3389/fendo.2026.1900593

**Published:** 2026-07-28

**Authors:** Dragana Puhalo Sladoje, Ilija Dragojević, Dejan Bokonjić, Lamija Zečević-Pašić, Olivera Čančar, Berina Hasanefendić, Bojana Kisić

**Affiliations:** 1 Faculty of Medicine Foča, University of East Sarajevo, Foča, Bosnia and Herzegovina; 2 Faculty of Medicine, University of Priština in Kosovska Mitrovica, Kosovska Mitrovica, Serbia; 3 Faculty of Medicine, University of Sarajevo, Sarajevo, Bosnia and Herzegovina; 4 University Hospital Foča, Foča, Bosnia and Herzegovina; 5 Clinical Center of the University of Sarajevo, Sarajevo, Bosnia and Herzegovina

**Keywords:** abdominal obesity, adipokines, adiponectin/leptin ratio, ghrelin, insulin resistance, metabolic syndrome, plasminogen activator inhibitor-1, young adults

## Abstract

**Background:**

Abdominal obesity in young adults may reflect a biologically active metabolic phenotype, even before full metabolic syndrome develops. However, how endocrine, adipokine, insulin-resistance, and fibrinolysis-related biomarkers vary across early metabolic phenotypes remains incompletely understood. This study examined whether selected circulating biomarkers differ among normal-weight controls, young adults with abdominal obesity, and those with metabolic syndrome.

**Methods:**

This cross-sectional secondary biomarker analysis included 175 young adults aged 19–21 years who were classified according to International Diabetes Federation criteria as controls (n = 106), abdominal obesity (n = 37), or metabolic syndrome (n = 32). Fasting insulin, HOMA-IR, cortisol, 25-hydroxyvitamin D, leptin, adiponectin, the adiponectin/leptin ratio (A/L ratio), ghrelin, and plasminogen activator inhibitor-1 (PAI-1) were analyzed. Between-group differences were assessed using nonparametric tests, ordered trends were evaluated across the phenotype gradient, sensitivity analyses were adjusted for sex, smoking status, and physical activity, and penalized logistic regression was used to explore biomarker associations with abdominal obesity and metabolic syndrome.

**Results:**

Insulin, HOMA-IR, cortisol, leptin, and PAI-1 showed positive ordered phenotype-related patterns, whereas ghrelin, adiponectin, and the A/L ratio showed negative ordered patterns. Vitamin D concentrations were lower in the abdominal obesity and metabolic syndrome groups than in controls. The strongest between-group effects were observed for PAI-1, cortisol, ghrelin, adiponectin, and the A/L ratio. Overall group effects remained significant after adjustment and false discovery rate correction. In mutually adjusted penalized models, the A/L ratio remained associated with abdominal obesity, whereas PAI-1 remained associated with metabolic syndrome.

**Conclusion:**

Young adults with abdominal obesity showed measurable endocrine, adipokine, insulin-resistance, and fibrinolysis-related alterations, including those who did not meet criteria for metabolic syndrome. The A/L ratio and PAI-1 may provide complementary information, with the A/L ratio more closely associated with abdominal obesity and PAI-1 more closely associated with metabolic syndrome in exploratory models. These findings support further evaluation of phenotype-based biomarker profiling for characterizing early cardiometabolic dysregulation in young adults and should be confirmed in larger, sex-balanced cohorts.

## 1 Introduction

Obesity has become one of the most important health challenges across the lifespan, and its increasing prevalence among children, adolescents, and young adults is particularly concerning. Recent global data suggest that more than 1 billion people were living with obesity in 2022, and excess body weight at younger ages is expected to contribute substantially to the future burden of cardiometabolic disease (1). This stage of life is especially important because excess body weight acquired early may persist into adulthood, prolonging exposure to metabolic and vascular risk factors and contributing to early cardiometabolic disturbances before overt disease develops (2).

Although body mass index (BMI) remains the most widely used screening tool in clinical practice and population studies, it has important limitations. BMI does not directly capture body composition, fat distribution, or individual differences related to sex and lean body mass (3, 4). Recent consensus statements emphasize that BMI remains useful for describing risk at the population level, but is less reliable for assessing individual risk unless interpreted together with other anthropometric and clinical measures (4).

In this context, measures of central adiposity, particularly waist circumference and waist-based ratios, may provide more clinically meaningful information because abdominal fat accumulation is more closely associated with cardiometabolic risk than BMI alone (5, 6). Therefore, when studying early metabolic deterioration in young adults, a phenotype-based classification that includes normal-weight controls, individuals with abdominal obesity, and individuals with metabolic syndrome may be more informative than grouping participants by BMI alone (5, 6).

The adverse cardiometabolic effects of obesity are increasingly understood through the lens of adipose tissue dysfunction, particularly in visceral fat. Adipose tissue is no longer regarded simply as a storage site for excess energy, but as an active endocrine and immune organ. Through the release of adipokines and other biologically active mediators, adipose tissue contributes to the regulation of energy balance, insulin sensitivity, inflammation, vascular function, and thrombosis (7, 8).

In obesity, expansion and remodeling of adipose tissue, especially in visceral depots, can promote chronic low-grade inflammation, disrupt adipokine secretion, and gradually worsen insulin resistance. Together, these changes contribute to the clustering of cardiometabolic risk factors (7–10). This suggests that early metabolic disturbances may be detectable through biomarker profiling even before clear clinical disease becomes evident.

Metabolic syndrome refers to a cluster of closely related abnormalities, including central obesity, elevated blood pressure, atherogenic dyslipidemia, and impaired glucose regulation. In young adults, the International Diabetes Federation (IDF) adult criteria provide a practical framework for classifying cardiometabolic phenotypes, requiring abdominal obesity as a core component together with additional metabolic abnormalities to define metabolic syndrome (11).

This approach is particularly useful because it distinguishes individuals with abdominal obesity who do not meet criteria for metabolic syndrome from those who already meet the diagnostic criteria. It therefore allows researchers to compare biomarker profiles across an ordered metabolic phenotype spectrum, from controls to abdominal obesity and metabolic syndrome, without implying longitudinal progression between categories.

A multidomain biomarker approach is appropriate in this setting because differences across metabolic phenotypes may involve more than insulin resistance alone. Alterations in adipokines, endocrine regulation, and fibrinolysis-related activity may already be present even when overt hyperglycemia is not prominent. Accordingly, fasting insulin and HOMA-IR can help identify early insulin resistance, while the combined assessment of adipokine-related, endocrine, and fibrinolysis-related markers may provide a more sensitive view of phenotype-related metabolic differences than conventional metabolic variables alone (9, 12, 13).

Although many studies have examined individual biomarkers in relation to obesity and metabolic syndrome, fewer have evaluated endocrine, adipokine, and fibrinolysis-related markers together in young adults (14). This combined approach may be particularly useful for detecting biological dysregulation in individuals with abdominal obesity who do not yet meet full criteria for metabolic syndrome. The aim of this cross-sectional study was to examine whether endocrine, adipokine, insulin-resistance, vitamin D, ghrelin, and fibrinolysis-related biomarkers differed across three young-adult phenotypes: normal-weight controls, abdominal obesity without metabolic syndrome, and metabolic syndrome. We hypothesized that these biomarkers would show phenotype-dependent differences across the ordered control, abdominal obesity, and metabolic syndrome groups.

## 2 Materials and methods

### 2.1 Study design and participants

This cross-sectional study was conducted in March 2014 during routine systematic health examinations of first- and second-year students at the Faculty of Medicine in Foča, University of East Sarajevo. Clinical questionnaire data, anthropometric and blood pressure measurements, and fasting blood sampling were obtained during the same examination period. The final analytical sample consisted of 175 participants of both sexes, aged 19–21 years.

Baseline information was collected from medical history and a structured questionnaire and included sex, age, cigarette smoking, physical activity, and self-reported chronic diseases. All participants were informed about the study objectives and provided written informed consent before enrollment. The study protocol was approved by the Ethics Committee of the Faculty of Medicine in Foča, University of East Sarajevo (Decision No. 260/2), and was conducted in accordance with the Declaration of Helsinki.

The present analysis was based on the same cohort as an earlier publication, which reported anthropometric characteristics, blood pressure, lipid parameters, and inflammatory/apolipoprotein markers (15). The current study examined a distinct endocrine, adipokine, insulin-resistance, vitamin D, ghrelin, and fibrinolysis-related biomarker panel. Because the analysis was based on available data from this predefined analytical sample, no formal *a priori* sample size calculation was performed, and regression analyses were interpreted as exploratory.

### 2.2 Eligibility criteria

Participants were eligible for inclusion if they were older than 18 years, were first- or second-year students undergoing routine systematic health examination, and provided written informed consent.

Participants were excluded if they had a previously diagnosed cardiovascular, hepatic, renal, central nervous system, endocrine, or other chronic systemic disease, or if they used medications that could affect glucose or lipid metabolism.

To minimize the potential influence of acute inflammation on biomarker levels, the analytical sample was restricted to participants with serum high-sensitivity C-reactive protein (hsCRP) concentrations below 10 mg/L.

### 2.3 Anthropometric and blood pressure measurements

Body height was measured to the nearest 0.1 cm using a Martin anthropometer, with participants standing barefoot in the Frankfurt plane, according to World Health Organization recommendations (16). Body weight was measured to the nearest 0.1 kg using a calibrated medical decimal scale. Body mass index (BMI) was calculated as body weight in kilograms divided by height in meters squared (kg/m²).

Waist and hip circumferences were measured in the standing position using a flexible measuring tape and were recorded to the nearest 0.1 cm. Waist circumference was measured at the midpoint between the lower costal margin and the iliac crest along the midaxillary line. Hip circumference was measured at the level of the greater trochanters. Waist-to-hip ratio (WHR) was calculated by dividing waist circumference by hip circumference.

Systolic and diastolic blood pressure were measured using a Riva–Rocci sphygmomanometer after participants had rested in a seated position for 5 minutes. Measurements were taken on the left arm, positioned at heart level, and recorded in mmHg.

### 2.4 Blood sampling and laboratory analyses

Laboratory analyses were performed at the Department of Medical Biochemistry, University Hospital Foča. Peripheral venous blood samples were collected in the morning before 10:00 a.m. after a 12-hour overnight fast, during routine systematic health examinations conducted in March 2014. Venipuncture was performed using a vacuum blood collection system. Blood was collected into tubes containing a clot activator for serum separation and into citrate tubes for plasma isolation.

Samples were processed within 30 minutes of collection and centrifuged at 2500 rpm for 15 minutes in a refrigerated centrifuge at 4 °C. Serum and plasma were separated into aliquots and stored continuously at −80 °C for approximately three months until biomarker analysis. Immediately before measurement, aliquots were thawed once at room temperature and gently mixed before analysis. No repeated freeze–thaw cycles were allowed.

Before analysis, samples were checked according to standard preanalytical quality-control procedures for visible hemolysis, lipemia, turbidity, insufficient volume, improper labeling, and clotting in anticoagulated tubes. No sample included in the final analytical dataset was excluded because of visible preanalytical problems.

All laboratory analyses were performed according to the manufacturers’ instructions, using routine internal quality-control procedures throughout the study.

Fasting glucose concentrations were measured in mmol/L on an Architect c4000 analyzer (Abbott, USA) using automated spectrophotometric methods and commercial reagents. Fasting insulin concentrations were measured in µIU/mL by chemiluminescent microparticle immunoassay on an Architect i1000 analyzer (Abbott, USA). Insulin resistance was estimated using the homeostasis model assessment of insulin resistance (HOMA-IR), calculated as:

HOMA-IR = fasting glucose (mmol/L) × fasting insulin (µIU/mL)/22.5 (17).

Triacylglycerols (TAG), high-density lipoprotein cholesterol (HDL-C), low-density lipoprotein cholesterol (LDL-C), and very-low-density lipoprotein cholesterol (VLDL-C) were measured on an Architect c4000 analyzer (Abbott, USA) using automated spectrophotometric methods.

Serum total adiponectin and leptin concentrations, as well as plasma plasminogen activator inhibitor-1 (PAI-1) concentrations, were measured by sandwich enzyme-linked immunosorbent assay (ELISA) (R&D Systems, Inc., USA). Serum total ghrelin concentrations were measured by sandwich ELISA (DRG International, Inc., USA). Serum 25-hydroxyvitamin D [25(OH)D] and cortisol concentrations were measured by chemiluminescent microparticle immunoassay on an Architect i1000 analyzer (Abbott, USA), according to the manufacturers’ instructions. The adiponectin/leptin (A/L) ratio was calculated for each participant using the following equation: A/L ratio = adiponectin (ng/mL)/leptin (pg/mL).

### 2.5 Definition of study groups

Participants were classified into three groups according to IDF criteria (11). The metabolic syndrome (MetS) group included participants with increased waist circumference, defined as ≥94 cm in males or ≥80 cm in females, together with at least two additional metabolic abnormalities: triacylglycerols (TAG) >1.70 mmol/L, HDL-C <1.03 mmol/L in males or <1.29 mmol/L in females, fasting glucose ≥5.6 mmol/L, systolic blood pressure ≥130 mmHg, and/or diastolic blood pressure ≥85 mmHg.

The abdominal obesity (AO) group included participants with increased waist circumference according to the same sex-specific cut-off values, but who did not meet the criteria for MetS. The control group included participants with normal body weight, normal waist circumference, and no cardiometabolic abnormalities according to the above IDF components.

### 2.6 Statistical analysis

All analyses were performed using IBM SPSS Statistics for Windows, version 26.0 (IBM Corp., Armonk, NY, USA), with the STATS FIRTHLOG extension command used for Firth penalized logistic regression.

Categorical variables were presented as numbers and percentages, while continuous variables were presented as medians and interquartile ranges (IQRs). Differences in categorical variables between groups were assessed using the chi-square test, with Monte Carlo exact significance when expected cell counts were small. Because the analyzed continuous variables were not normally distributed, between-group comparisons were performed using the Kruskal–Wallis test, followed by Bonferroni-adjusted *post hoc* pairwise comparisons when appropriate. Overall between-group effect sizes for Kruskal–Wallis tests were estimated using epsilon-squared (ϵ²).

Because the study groups represented a predefined ordered metabolic gradient from controls to abdominal obesity (AO) and metabolic syndrome (MetS), Jonckheere–Terpstra tests were used to assess monotonic trends across groups. For each biomarker or index, the standardized test statistic (Z), two-sided p-value for trend, and effect size expressed as r = Z/√N were reported.

Adjusted sensitivity analyses were performed using rank-based general linear models, with rank-transformed biomarker values as dependent variables, study group as a fixed factor, and sex, smoking status, and physical activity as covariates. The Benjamini–Hochberg false discovery rate procedure was applied across the biomarker panel.

Because sex distribution differed across phenotype groups, the main Kruskal–Wallis comparisons were also repeated in female participants as a supportive sensitivity analysis, with false discovery rate correction applied across biomarkers. This analysis was interpreted cautiously because of the reduced subgroup size.

Correlations between BMI and the analyzed biomarkers and indices were examined using Spearman’s rank correlation coefficient (ρ). Adjusted correlations were estimated after accounting for sex, smoking status, and physical activity using rank-transformed variables.

Exploratory Firth penalized logistic regression models were used to examine the associations of standardized biomarker values with AO and MetS relative to controls, with adjustment for sex, smoking status, and physical activity. Separate single-biomarker models were fitted for each biomarker, followed by compact mutually adjusted models including PAI-1 and the A/L ratio.

All tests were two-sided, and p < 0.05 was considered statistically significant.

## 3 Results

### 3.1 Participant characteristics and study design

**Figure 1:**
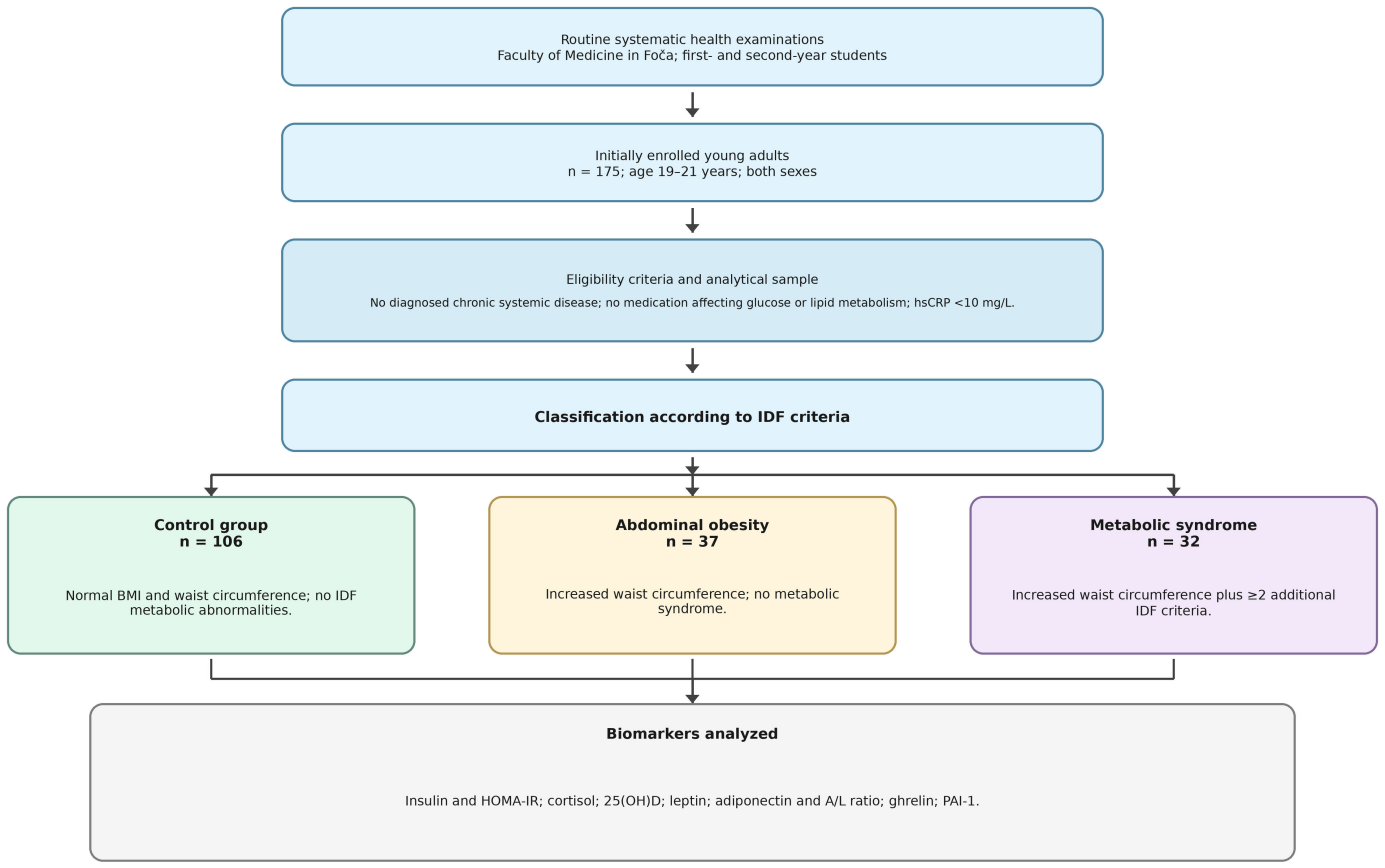
Study design and metabolic phenotype classification. The figure summarizes participant enrollment, eligibility criteria, IDF-based group classification, and the biomarker panel analyzed in the present study. The analytical sample included 175 young adults aged 19–21 years without diagnosed chronic systemic disease, without medication use affecting glucose or lipid metabolism, and with hsCRP <10 mg/L. Participants were classified according to IDF criteria as controls (n = 106), abdominal obesity (AO; n = 37), or metabolic syndrome (MetS; n = 32). AO was defined as increased waist circumference (≥94 cm in males or ≥80 cm in females) without metabolic syndrome. MetS was defined as increased waist circumference plus at least two additional IDF criteria: TAG >1.70 mmol/L, reduced HDL-C, fasting glucose ≥5.6 mmol/L, SBP ≥130 mmHg, and/or DBP ≥85 mmHg. The analyzed biomarkers included insulin, HOMA-IR, cortisol, 25(OH)D, leptin, adiponectin, the adiponectin/leptin ratio, ghrelin, and PAI-1. AO, abdominal obesity; MetS, metabolic syndrome; IDF, International Diabetes Federation; hsCRP, high-sensitivity C-reactive protein; TAG, triacylglycerols; HDL-C, high-density lipoprotein cholesterol; SBP, systolic blood pressure; DBP, diastolic blood pressure; HOMA-IR, homeostasis model assessment of insulin resistance; PAI-1, plasminogen activator inhibitor-1.

A total of 175 young adults were classified according to International Diabetes Federation (IDF) criteria into three groups: controls (n = 106), abdominal obesity (AO; n = 37), and metabolic syndrome (MetS; n = 32). The study design and group classification are shown in **Figure 1**.

Sex distribution differed significantly across the groups. Female participants were more frequent in the control and AO groups, whereas male participants predominated in the MetS group. Smoking status was comparable between groups, whereas regular physical activity differed significantly (**Table 1**).

**Table 1:** Participant characteristics across study groups.

Variable	Control (n = 106)	AO (n = 37)	MetS (n = 32)	p (overall)
Sex, n (%)				<0.001
Female	74 (70)	33 (89)	15 (47)	
Male	32 (30)	4 (11)	17 (53)	
Smoking, n (%)				0.310
No	95 (90)	29 (78)	29 (91)	
Yes	11 (10)	8 (22)	3 (9)	
Physical activity, n (%)				0.042
No	49 (46)	26 (70)	17 (53)	
Yes	57 (54)	11 (30)	15 (47)	

Data are presented as n (%). Between-group differences were assessed using the chi-square test. For smoking status, Monte Carlo exact significance was applied because of small expected cell counts. AO, abdominal obesity; MetS, metabolic syndrome.

### 3.2 Endocrine, adipokine, and fibrinolysis-related biomarkers across metabolic phenotypes

Endocrine, adipokine, insulin-resistance, vitamin D, ghrelin, and fibrinolysis-related biomarkers differed significantly across the three metabolic phenotype groups (**Table 2**). Bonferroni-adjusted *post hoc* differences are indicated by superscript letters in **Table 2**.

**Table 2:** Endocrine, adipokine, insulin-resistance, vitamin D, ghrelin, and PAI-1 biomarkers across metabolic phenotype groups.

Variable	Control median (IQR)	AO median (IQR)	MetS median (IQR)	Kruskal–Wallis p	ϵ²
Insulin (µIU/mL)	7.50 (6.50–9.03) **^a^**	8.70 (7.05–12.85) **^b^**	16.90 (12.73–18.30) **^c^**	<0.001	0.310
HOMA-IR	1.45 (1.18–1.70) **^a^**	1.60 (1.25–2.30) **^a^**	3.10 (2.33–3.78) **^b^**	<0.001	0.290
Adiponectin (ng/mL)	308.0 (276.8–327.2) **^a^**	130.3 (98.7–193.1) **^b^**	81.5 (59.5–124.5) **^b^**	<0.001	0.729
Leptin (pg/mL)	699.0 (641.3–794.3) **^a^**	2104.0 (1831.0–2291.0) **^b^**	2278.0 (2007.0–2537.8) **^b^**	<0.001	0.705
A/L ratio	0.4286 (0.3782–0.5170) **^a^**	0.0668 (0.0501–0.0819) **^b^**	0.0370 (0.0253–0.0594) **^b^**	<0.001	0.733
Ghrelin (pg/mL)	184.6 (177.3–189.6) **^a^**	138.9 (136.1–140.9) **^b^**	117.7 (111.9–120.1) **^c^**	<0.001	0.740
Cortisol (µg/dL)	10.90 (10.50–12.30) **^a^**	16.30 (15.55–17.20) **^b^**	20.65 (19.53–21.83) **^c^**	<0.001	0.744
Vitamin D (ng/mL)	23.10 (20.90–24.63) **^a^**	16.70 (14.90–19.80) **^b^**	20.20 (16.35–22.45) **^b^**	<0.001	0.276
PAI-1 (ng/mL)	10.90 (9.90–12.10) **^a^**	20.40 (17.65–21.90) **^b^**	28.90 (26.90–29.80) **^c^**	<0.001	0.758

Values are presented as median (interquartile range). Between-group differences were evaluated using the Kruskal–Wallis test. Effect size is reported as epsilon-squared (ϵ²), where higher values indicate a larger overall between-group effect. Different superscript letters within the same row indicate statistically significant Bonferroni-adjusted *post hoc* differences between groups (p < 0.05); groups sharing the same letter do not differ significantly. Detailed pairwise p-values are provided in **Supplementary Table S1** for transparency. C, control; AO, abdominal obesity; MetS, metabolic syndrome; HOMA-IR, homeostasis model assessment of insulin resistance; A/L ratio, adiponectin/leptin ratio; PAI-1, plasminogen activator inhibitor-1.

Insulin and HOMA-IR differed significantly across the study groups. Insulin increased stepwise from controls to AO and MetS, whereas HOMA-IR was higher in MetS than in both controls and AO, with no significant difference between controls and AO.

Cortisol, ghrelin, and PAI-1 showed the clearest graded differences across the ordered phenotype groups. Cortisol and PAI-1 were higher in AO than in controls and highest in MetS, whereas ghrelin was lower in AO than in controls and lowest in MetS.

Vitamin D, adiponectin, and the adiponectin/leptin ratio were lower in both AO and MetS than in controls, whereas leptin was higher in both AO and MetS than in controls. For these biomarkers, AO and MetS did not differ significantly from each other.

**Figure 2:**
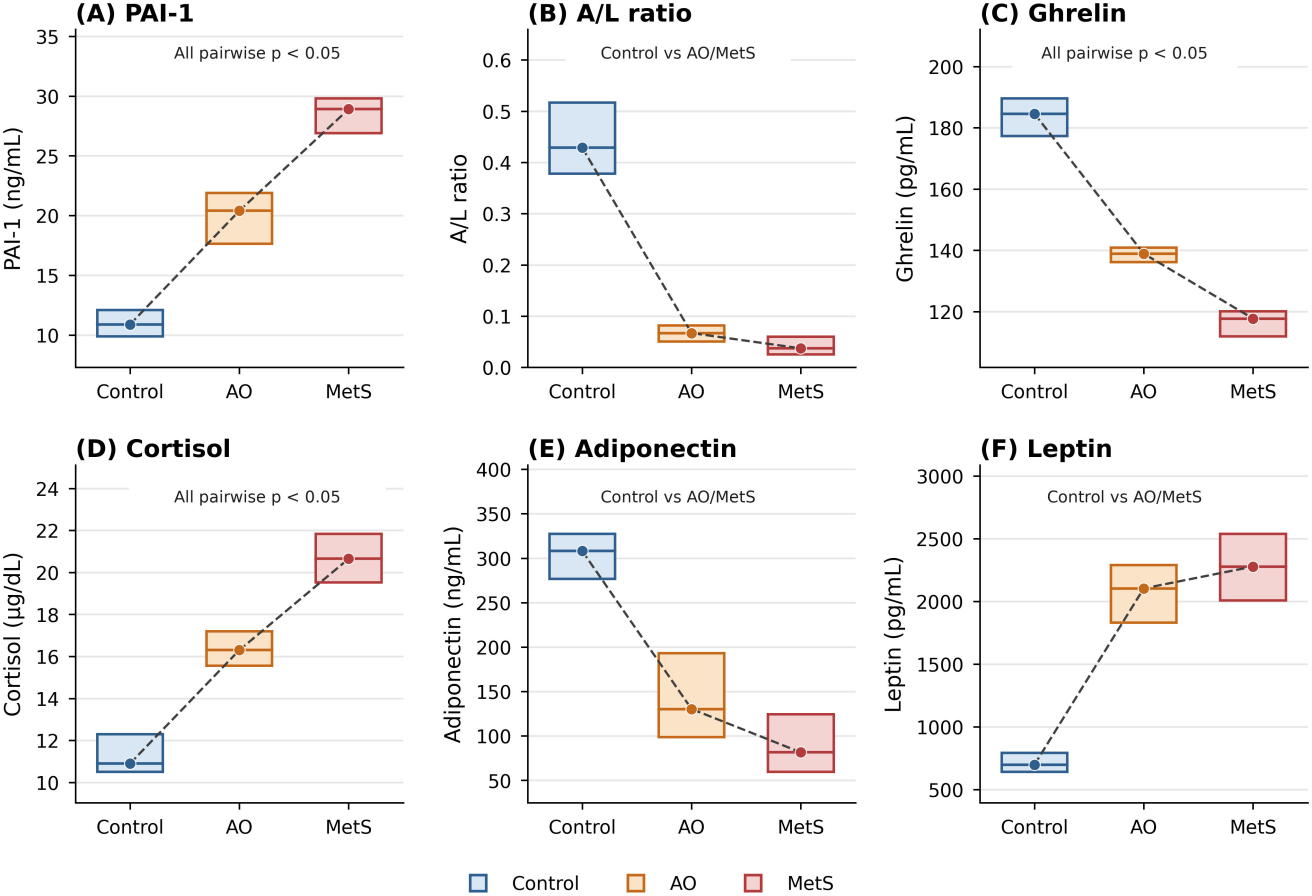
Distribution of selected endocrine, adipokine, and fibrinolysis-related biomarkers across metabolic phenotype groups. Box plots show concentrations of PAI-1 **(A)**, adiponectin/leptin ratio (A/L ratio) **(B)**, ghrelin **(C)**, cortisol **(D)**, adiponectin **(E)**, and leptin **(F)** in the control, abdominal obesity (AO), and metabolic syndrome (MetS) groups. Boxes represent the interquartile range, horizontal lines indicate medians, and colored circular markers connected by dashed lines represent group medians. Bonferroni-adjusted pairwise comparisons are indicated within each panel. All pairwise comparisons were significant for PAI-1, ghrelin, and cortisol. For the A/L ratio, adiponectin, and leptin, controls differed significantly from both AO and MetS, whereas AO and MetS did not differ significantly from each other. AO, abdominal obesity; MetS, metabolic syndrome; PAI-1, plasminogen activator inhibitor-1; A/L ratio, adiponectin/leptin ratio.

These key endocrine, adipokine, and fibrinolysis-related differences are illustrated in **Figure 2**. The largest between-group effect sizes were observed for PAI-1, cortisol, ghrelin, the adiponectin/leptin ratio, and adiponectin, followed closely by leptin.

### 3.3 Ordered biomarker trends and adjusted sensitivity analysis

**Figure 3:**
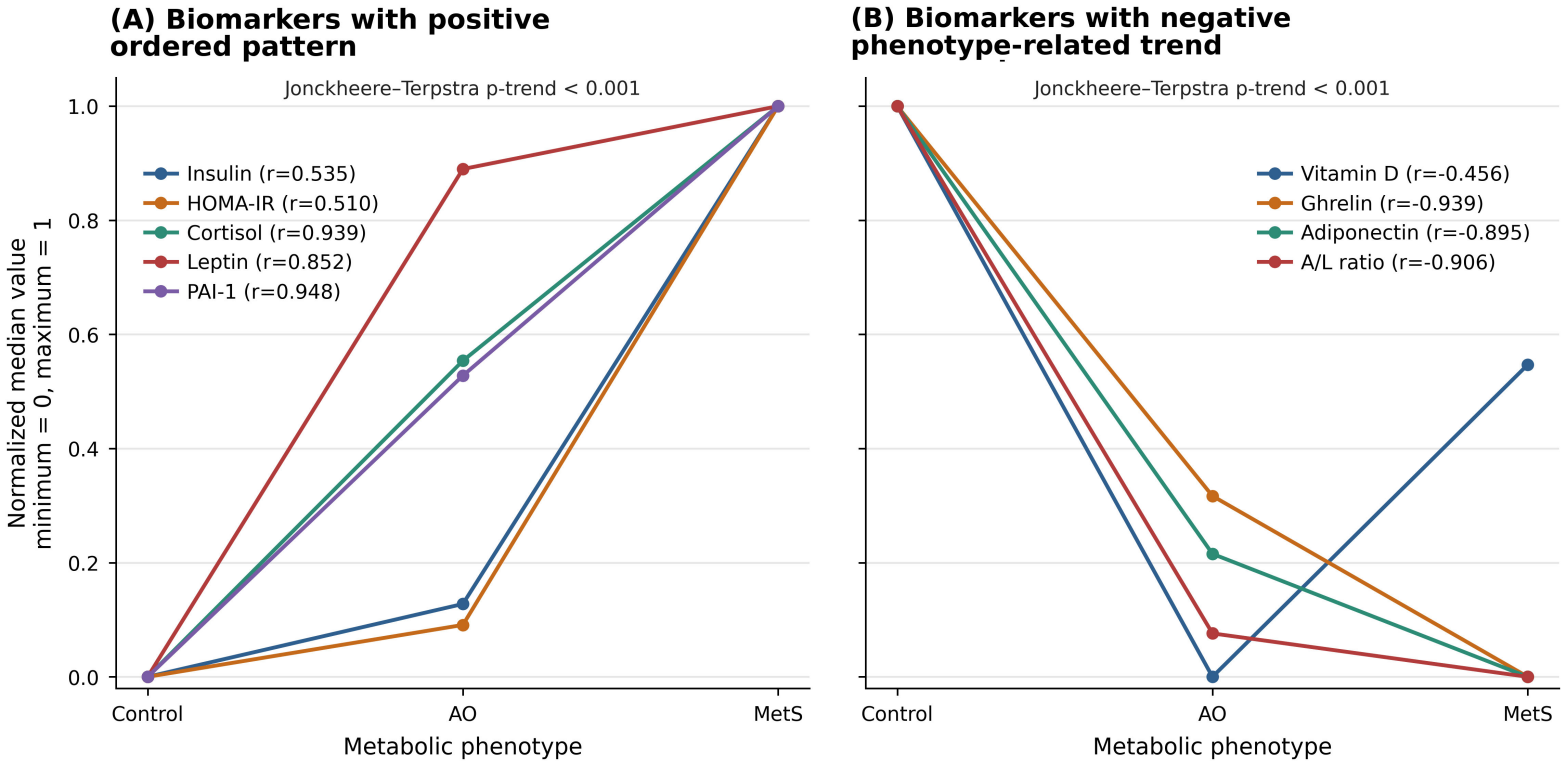
Ordered phenotype-related biomarker patterns across control, abdominal obesity, and metabolic syndrome groups. Lines show min–max normalized group medians, allowing biomarkers measured in different units to be displayed on the same scale. Panel **(A)** presents biomarkers with higher median values across the ordered phenotype sequence. Panel **(B)** presents biomarkers with lower phenotype-related values. For vitamin D, the overall Jonckheere–Terpstra trend was negative, but the descriptive pattern was not strictly stepwise because vitamin D was lowest in AO and higher in MetS, although both AO and MetS were lower than controls. Normalized values are shown for visualization only and were not used for statistical testing. Ordered patterns were assessed using the Jonckheere–Terpstra test across the predefined phenotype sequence: control → abdominal obesity (AO) → metabolic syndrome (MetS). The reported r values represent effect sizes calculated as Z/√N. AO, abdominal obesity; MetS, metabolic syndrome; HOMA-IR, homeostasis model assessment of insulin resistance; PAI-1, plasminogen activator inhibitor-1; A/L ratio, adiponectin/leptin ratio.

To assess whether the group differences shown in **Table 2** followed the predefined phenotype sequence, Jonckheere–Terpstra tests were performed across the control, AO, and MetS groups. Insulin, HOMA-IR, cortisol, leptin, and PAI-1 showed significant positive ordered patterns, whereas vitamin D, ghrelin, adiponectin, and the adiponectin/leptin ratio showed significant negative ordered patterns (**Figure 3**; **Supplementary Table 2**).

These analyses confirmed that the main biomarker differences observed in **Table 2** were not limited to overall between-group comparisons, but were also consistent with the predefined ordered phenotype sequence.

In adjusted sensitivity analyses using rank-transformed biomarker values, the overall group effect remained significant for all analyzed biomarkers after adjustment for sex, smoking status, and physical activity. The pattern of results was unchanged after Benjamini–Hochberg false discovery rate correction (**Supplementary Table 3**).

Because sex distribution differed significantly across groups, an additional female-only sensitivity analysis was performed. Female subgroup sizes were 74 controls, 33 participants with AO, and 15 participants with MetS. Despite the reduced sample size, all analyzed biomarkers remained significantly different across the three phenotype groups after Benjamini–Hochberg false discovery rate correction. The overall direction of median biomarker differences was consistent with the full-cohort analysis: insulin, HOMA-IR, leptin, cortisol, and PAI-1 were higher in metabolically unfavorable phenotypes, whereas adiponectin, the A/L ratio, ghrelin, and vitamin D were lower than in controls. Bonferroni-adjusted *post hoc* patterns were broadly consistent with the main analysis, although AO–MetS pairwise differences were attenuated for several biomarkers. This analysis was considered supportive rather than definitive because of the smaller number of female participants with MetS (**Supplementary Table 4**).

### 3.4 Supportive BMI–biomarker association analyses

**Figure 4:**
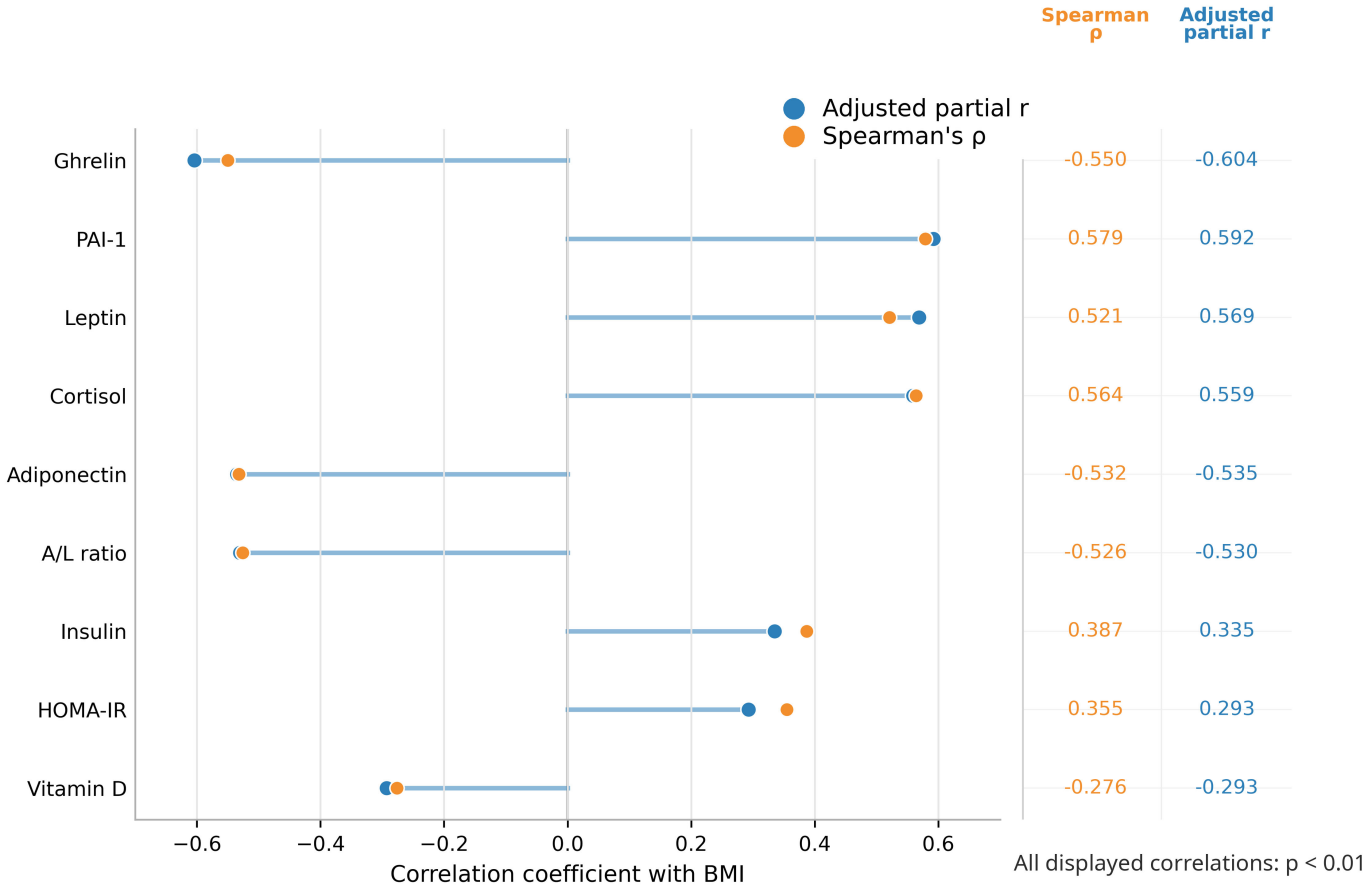
Associations between BMI and circulating biomarkers and derived indices. Horizontal lines are visual lollipop stems extending to zero and do not represent confidence intervals. The figure presents unadjusted Spearman’s correlation coefficients and adjusted partial correlations between BMI and selected biomarkers and derived indices. Adjusted partial correlations were calculated after rank transformation and adjustment for sex, smoking status, and physical activity. The p-value threshold is indicated directly in the figure; all displayed correlations were statistically significant at p < 0.01, two-sided. BMI, body mass index; HOMA-IR, homeostasis model assessment of insulin resistance; A/L ratio, adiponectin/leptin ratio; PAI-1, plasminogen activator inhibitor-1.

To examine whether phenotype-related biomarker differences were also reflected in adiposity-related correlations, BMI–biomarker correlations were assessed (**Figure 4**). Higher BMI was associated with lower adiponectin, adiponectin/leptin ratio, ghrelin, and vitamin D, and with higher leptin, insulin, HOMA-IR, cortisol, and PAI-1. These correlations were largely maintained after adjustment for sex, smoking status, and physical activity. The corresponding p-value threshold is indicated in **Figure 4**. Overall, these BMI–biomarker correlations were consistent with the phenotype-based findings, but should be interpreted as supportive cross-sectional analyses rather than as independent evidence of prediction or progression.

### 3.5 Exploratory penalized logistic regression models

Because ordinary logistic regression showed separation-related instability for several biomarkers, Firth penalized logistic regression was used. These analyses were interpreted as exploratory and supportive association analyses rather than as precise estimates of effect size or individual risk prediction.

In adjusted single-biomarker penalized logistic regression models, PAI-1, the adiponectin/leptin ratio, ghrelin, and cortisol were each significantly associated with both abdominal obesity and metabolic syndrome compared with controls (**Supplementary Table 5**). Higher PAI-1 and cortisol concentrations were associated with higher odds of belonging to the AO or MetS groups, whereas higher ghrelin concentrations and higher adiponectin/leptin ratio values were associated with lower odds.

Because several models produced very large odds ratios with wide confidence intervals, the magnitude of these estimates should be interpreted with caution. Therefore, the regression models should be viewed primarily as supportive evidence for the direction and consistency of biomarker–phenotype associations observed in the group comparisons, ordered phenotype-related analyses, and BMI–biomarker association analyses.

In the exploratory combined model including PAI-1 and the adiponectin/leptin ratio simultaneously, the adiponectin/leptin ratio remained associated with abdominal obesity, whereas PAI-1 remained associated with metabolic syndrome (**Table 3**). These findings suggest that the two biomarkers may provide complementary information, with the adiponectin/leptin ratio more closely associated with abdominal obesity and PAI-1 more closely associated with metabolic syndrome in mutually adjusted exploratory models.

**Table 3:** Exploratory combined penalized logistic regression model including PAI-1 and the adiponectin/leptin ratio for abdominal obesity and metabolic syndrome compared with controls.

Comparison	Predictor	Adjusted OR (95% CI) per 1-SD increase	p value
Abdominal obesity vs controls (n = 143)	PAI-1	2.34 (0.0602 to 90.6)	0.649
	A/L ratio	0.0135 (4.72 × 10–4 to 0.387)	0.012
Metabolic syndrome vs controls (n = 138)	PAI-1	20.6 (1.43 to 298)	0.026
	A/L ratio	0.663 (0.0476 to 9.24)	0.760

Data are presented as odds ratios (ORs) with 95% confidence intervals (CIs) per 1-SD increase in the biomarker or derived index. The combined model included PAI-1 and the A/L ratio simultaneously and was adjusted for sex, physical activity, and smoking status. Penalized logistic regression was used because of separation-related instability in ordinary logistic regression models. These models were interpreted as exploratory association analyses rather than as validated prediction models. A/L ratio, adiponectin/leptin ratio; AO, abdominal obesity; CI, confidence interval; MetS, metabolic syndrome; OR, odds ratio; PAI-1, plasminogen activator inhibitor-1.

## 4 Discussion

### 4.1 Interpretation of the main findings

In this cross-sectional study of young adults, endocrine, adipokine, insulin-resistance, vitamin D, ghrelin, and fibrinolysis-related biomarkers differed across IDF-defined metabolic phenotypes. Participants with abdominal obesity already showed an unfavorable biomarker profile compared with controls, whereas participants with metabolic syndrome generally showed more pronounced alterations. These findings suggest that central adiposity in young adults may be accompanied by measurable biological differences even before the full metabolic syndrome phenotype is present, consistent with the recognized cardiometabolic relevance of abdominal obesity (18). However, because of the cross-sectional design, these differences should be interpreted as phenotype-associated patterns rather than evidence of longitudinal progression.

The biomarker pattern was not uniform across all markers. Leptin, adiponectin, the A/L ratio, and vitamin D mainly separated participants with abdominal obesity from controls, whereas insulin, cortisol, ghrelin, and PAI-1 showed a more gradual pattern across the control, AO, and MetS groups. This distinction may suggest that some biomarkers are more closely related to adipose-endocrine disturbance, whereas others may better reflect broader metabolic-endocrine and fibrinolysis-related dysregulation. In particular, the A/L ratio may represent an integrated marker of adipose tissue dysfunction and obesity-related cardiometabolic risk, whereas PAI-1 may reflect impaired fibrinolytic balance associated with metabolic syndrome (19, 20).

### 4.2 Adipokine imbalance and insulin-resistance patterns

The adipokine findings suggest an unfavorable adipose-tissue-related profile in participants with AO and MetS, characterized by higher leptin and lower adiponectin and A/L ratio values than in controls. This direction is biologically plausible, because leptin generally increases with adiposity, whereas adiponectin tends to decrease in states of visceral adiposity, adipose tissue dysfunction, and insulin resistance (8, 18, 21, 22). However, adipokine concentrations are influenced by several factors, including sex, fat distribution, inflammatory status, hormonal factors, and assay-related differences. Therefore, the present findings should be interpreted as phenotype-associated differences within this cohort rather than as a universal adipokine pattern.

The A/L ratio may be useful because it integrates two adipokine signals that often move in opposite directions with increasing adiposity. In the present study, the A/L ratio showed one of the largest between-group differences and was lower in both AO and MetS than in controls. Previous studies have suggested that the A/L ratio may reflect adipose tissue dysfunction, adipose inflammation, insulin resistance, and obesity-related cardiometabolic risk more comprehensively than either adiponectin or leptin alone (20, 22–24). Nevertheless, its interpretation remains exploratory, and its clinical or predictive value in young adults requires confirmation in larger and more balanced cohorts.

Insulin-related findings provided additional evidence of metabolic disturbance, but the pattern was not identical for fasting insulin and HOMA-IR. Fasting insulin increased stepwise across controls, AO, and MetS, whereas HOMA-IR mainly separated MetS from the other two groups. This may indicate that compensatory hyperinsulinemia is detectable in abdominal obesity before more evident insulin-resistance clustering becomes apparent. This interpretation is consistent with studies suggesting that fasting insulin and insulin-resistance indices may identify early metabolic disturbance in younger individuals more sensitively than fasting glucose alone (25–27). However, because fasting glucose was also a diagnostic component of MetS and the study was cross-sectional, these findings should not be interpreted as evidence of progression from AO to MetS.

### 4.3 Broader endocrine and fibrinolysis-related signals

Cortisol, ghrelin, vitamin D, and PAI-1 provided additional information beyond adipokines and insulin resistance. Cortisol increased across the phenotype groups, which may be compatible with links between glucocorticoid activity, visceral adiposity, insulin resistance, and metabolic syndrome (28–30). However, circulating cortisol is influenced by sampling conditions, stress, circadian rhythm, and tissue-specific glucocorticoid metabolism, and previous studies have not uniformly shown higher basal cortisol in obesity-related states (30, 31). Therefore, the cortisol findings should be interpreted cautiously.

Ghrelin showed the opposite pattern, with lower concentrations in AO and MetS than in controls. This direction is broadly consistent with reports linking lower ghrelin levels with obesity-related metabolic disturbance and components of metabolic syndrome (32–34). However, ghrelin interpretation is complex because circulating concentrations depend on nutritional status, sample handling, assay type, and whether total, acylated, or des-acyl ghrelin is measured (34). In the present study, total ghrelin was measured, which should be considered when comparing these findings with studies assessing specific ghrelin fractions.

Vitamin D concentrations were lower in AO and MetS than in controls, but did not clearly distinguish AO from MetS. This pattern may suggest that reduced 25(OH)D status is more closely related to abdominal obesity itself than to the full metabolic syndrome phenotype in this young cohort. Previous studies have reported inverse associations between 25(OH)D concentrations and BMI, abdominal obesity, insulin resistance, and metabolic syndrome parameters (35–37). Nevertheless, vitamin D status is influenced by sunlight exposure, seasonality, diet, supplementation, physical activity, and adipose tissue sequestration, which were not fully assessed in this study.

PAI-1 showed one of the clearest graded patterns across the phenotype groups. This finding supports the concept that early metabolic deterioration may also involve fibrinolysis-related disturbance, not only adipokine imbalance or insulin resistance. Previous studies have linked PAI-1 with abdominal obesity, visceral adiposity, insulin resistance, endothelial dysfunction, and metabolic syndrome severity (19, 38). In the present study, PAI-1 was strongly associated with BMI and remained associated with MetS in the exploratory mutually adjusted model, suggesting that it may reflect a more advanced hemostatic component of the metabolic syndrome phenotype. This interpretation remains hypothesis-generating and requires confirmation in longitudinal studies.

### 4.4 Phenotype-based classification and exploratory models

The BMI-correlation analyses supported the phenotype-based findings by showing that increasing adiposity was associated with a less favorable biomarker profile. However, BMI alone cannot fully describe metabolic heterogeneity, because it does not capture fat distribution, central adiposity, or adipose tissue function (3, 4). Waist circumference and central adiposity measures provide additional cardiometabolic information beyond BMI, particularly because abdominal fat accumulation is more closely linked to visceral adiposity and metabolic risk (5). In this study, classification into controls, AO, and MetS provided an additional framework for interpreting biomarker differences beyond BMI alone.

The exploratory mutually adjusted models suggested that the A/L ratio and PAI-1 may reflect partly different aspects of early metabolic dysregulation. The A/L ratio was more closely associated with AO, which may be consistent with its role as an integrated adipokine marker of adipose tissue dysfunction (20, 22). In contrast, PAI-1 was more closely associated with MetS, suggesting that fibrinolysis-related disturbance may become more evident when abdominal obesity is accompanied by additional metabolic abnormalities (19, 38). This interpretation remains hypothesis-generating, because the models were exploratory, precision was limited, and external validation was not performed. Therefore, the A/L ratio and PAI-1 should be regarded as complementary research markers rather than as validated clinical screening or prediction tools.

### 4.5 Strengths and limitations

This study has several strengths. It used a clinically meaningful phenotype classification based on IDF criteria, allowing comparison of young adults with normal weight, abdominal obesity without metabolic syndrome, and metabolic syndrome (11). In addition, the study assessed a multidomain biomarker panel covering insulin resistance, adipokines, endocrine markers, vitamin D status, ghrelin, and fibrinolysis-related activity. The statistical approach combined nonparametric group comparisons, ordered-trend analyses, adjusted sensitivity analyses, correction for multiple testing, BMI-correlation analyses, and exploratory penalized regression models.

Several limitations should also be acknowledged. The cross-sectional design does not allow causal or temporal inference, and the findings cannot determine whether individuals with abdominal obesity will later develop metabolic syndrome. The study was conducted at a single center in a relatively homogeneous student-based cohort, which may limit generalizability to broader young-adult populations. In addition, the subgroup structure was uneven, particularly with respect to sex distribution, and residual sex-related confounding cannot be excluded despite adjusted and female-only sensitivity analyses.

Residual confounding by unmeasured factors also remains possible, including diet, sleep, menstrual cycle phase, oral contraceptive use, stress, sunlight exposure, vitamin D supplementation, and seasonality. These factors may be relevant for several of the analyzed biomarkers. Finally, the penalized regression models were exploratory and affected by limited precision; therefore, the A/L ratio and PAI-1 findings should be interpreted as hypothesis-generating rather than as validated diagnostic or predictive evidence. Larger, more diverse, sex-balanced, and longitudinal studies are needed to confirm these findings.

## 5 Conclusion

In this cross-sectional study of young adults, abdominal obesity and metabolic syndrome were associated with distinct endocrine, adipokine, insulin-resistance, ghrelin, vitamin D, and fibrinolysis-related biomarker patterns. Participants with abdominal obesity already showed measurable biomarker alterations compared with controls, whereas metabolic syndrome was generally associated with more pronounced changes. The A/L ratio and PAI-1 may provide complementary research information, with the A/L ratio more closely related to abdominal obesity and PAI-1 more closely related to metabolic syndrome in exploratory models. These findings should be interpreted as phenotype-associated and hypothesis-generating, and require confirmation in larger, sex-balanced, and longitudinal cohorts.

## Data Availability

The datasets presented in this article are not readily available because of ethical and privacy restrictions related to human-participant data. De-identified data supporting the conclusions of this article may be made available by the corresponding author upon reasonable request and in accordance with applicable ethical, institutional, and privacy requirements. Requests to access the datasets should be directed to Dragana Puhalo Sladoje, sladojedragana@gmail.com.

## References

[1] NCD Risk Factor Collaboration (NCD-RisC) . Worldwide trends in underweight and obesity from 1990 to 2022: a pooled analysis of 3663 population-representative studies with 222 million children, adolescents, and adults. Lancet. (2024) 403:1027–50. doi: 10.1016/S0140-6736(23)02750-2 38432237 PMC7615769

[2] Valerio G Di Bonito P Calcaterra V Cherubini V Corica D De Sanctis L . Cardiometabolic risk in children and adolescents with obesity: a position paper of the Italian Society for Pediatric Endocrinology and Diabetology. Ital J Pediatr. (2024) 50:205. doi: 10.1186/s13052-024-01767-x 39380079 PMC11463079

[3] Wu Y Li D Vermund SH . Advantages and limitations of the body mass index (BMI) to assess adult obesity. Int J Environ Res Public Health. (2024) 21:757. doi: 10.3390/ijerph21060757 38929003 PMC11204233

[4] Rubino F Cummings DE Eckel RH Cohen RV Wilding JPH Brown WA . Definition and diagnostic criteria of clinical obesity. Lancet Diabetes Endocrinol. (2025) 13:221–62. doi: 10.1016/S2213-8587(24)00316-4 39824205 PMC11870235

[5] Ross R Neeland IJ Yamashita S Shai I Seidell J Magni P . Waist circumference as a vital sign in clinical practice: a consensus statement from the IAS and ICCR Working Group on Visceral Obesity. Nat Rev Endocrinol. (2020) 16:177–89. doi: 10.1038/s41574-019-0310-7 32020062 PMC7027970

[6] Shetty S Suvarna R Bhattacharya S Seetharaman K . Visceral adiposity and cardiometabolic risk: clinical insights and assessment. Cardiol Rev. (2025). doi: 10.1097/CRD.0000000000000984 40622164

[7] Hemat Jouy S Mohan S Scichilone G Mostafa A Mahmoud AM . Adipokines in the crosstalk between adipose tissues and other organs: implications in cardiometabolic diseases. Biomedicines. (2024) 12:2129. doi: 10.3390/biomedicines12092129 39335642 PMC11428859

[8] Blüher M . Understanding adipose tissue dysfunction. J Obes Metab Syndr. (2024) 33:275–88. doi: 10.7570/jomes24013 39734091 PMC11704217

[9] Sunita S Ghozali M Rizki Akbar M Nugrahani AD Rakhmat II Fatimah SN . Recent insights into circulating adipokines in obesity: systematic review and meta-analysis. Obes Sci Pract. (2026) 12:e70113. doi: 10.1002/osp4.70113 41573104 PMC12820794

[10] Lee MJ Kim J . The pathophysiology of visceral adipose tissues in cardiometabolic diseases. Biochem Pharmacol. (2024) 222:116116. doi: 10.1016/j.bcp.2024.116116 38460909 PMC11407912

[11] Zimmet P Alberti KGMM Kaufman F Tajima N Silink M Arslanian S . The metabolic syndrome in children and adolescents: an IDF consensus report. Pediatr Diabetes. (2007) 8:299–306. doi: 10.1111/j.1399-5448.2007.00271.x 17850473

[12] Gutch M Kumar S Razi SM Gupta KK Gupta A . Assessment of insulin sensitivity/resistance. Indian J Endocrinol Metab. (2015) 19:160–4. doi: 10.4103/2230-8210.146874 25593845 PMC4287763

[13] Tilg H Ianiro G Gasbarrini A Adolph TE . Adipokines: masterminds of metabolic inflammation. Nat Rev Immunol. (2025) 25:250–65. doi: 10.1038/s41577-024-01103-8 39511425

[14] Schutte AE Huisman HW Schutte R van Rooyen JM Malan L Malan NT . Aging influences the level and functions of fasting plasma ghrelin levels: the POWIRS-Study. Regul Pept. (2007) 139:65–71. doi: 10.1016/j.regpep.2006.10.006 17113660

[15] Sladoje DP Kisić B Mirić D . The monitoring of protein markers of inflammation and serum lipid concentration in obese subjects with metabolic syndrome. J Med Biochem. (2017) 36:366–74. doi: 10.1515/jomb-2017-0009 30581334 PMC6294090

[16] World Health Organization . Measuring obesity. Classification and description of anthropometric data. Report on a WHO consultation on the epidemiology of obesity. Copenhagen: WHO Regional Office for Europe. (1989).

[17] Wallace TM Levy JC Matthews DR . Use and abuse of HOMA modeling. Diabetes Care. (2004) 27:1487–95. doi: 10.2337/diacare.27.6.1487 15161807

[18] Després JP Lemieux I . Abdominal obesity and metabolic syndrome. Nature. (2006) 444:881–7. doi: 10.1038/nature05488 17167477

[19] Alessi MC Juhan-Vague I . PAI-1 and the metabolic syndrome: links, causes, and consequences. Arterioscler Thromb Vasc Biol. (2006) 26:2200–7. doi: 10.1161/01.ATV.0000242905.41404.68 16931789

[20] Frühbeck G Catalán V Rodríguez A Gómez-Ambrosi J . Adiponectin-leptin ratio: a promising index to estimate adipose tissue dysfunction. Relation with obesity-associated cardiometabolic risk. Adipocyte. (2018) 7:57–62. doi: 10.1080/21623945.2017.1402151 29205099 PMC5915018

[21] Gherlan I Vladoiu S Alexiu F Giurcaneanu M Oros S Brehar A . Adipocytokine profile and insulin resistance in childhood obesity. Maedica (Buchar). (2012) 7 (3):205–13. PMC3566883 23400230

[22] Frühbeck G Catalán V Rodríguez A Ramírez B Becerril S Salvador J . Adiponectin-leptin ratio is a functional biomarker of adipose tissue inflammation. Nutrients. (2019) 11:454. doi: 10.3390/nu11020454 30813240 PMC6412349

[23] Frithioff-Bøjsøe C Lund MAV Lausten-Thomsen U Hedley PL Pedersen O Christiansen M . Leptin, adiponectin, and their ratio as markers of insulin resistance and cardiometabolic risk in childhood obesity. Pediatr Diabetes. (2020) 21:194–202. doi: 10.1111/pedi.12964 31845423

[24] Agostinis-Sobrinho C Vicente SECF Norkiene S Rauckienė-Michaelsson A Kievisienė J Dubey VP . Is the leptin/adiponectin ratio a better diagnostic biomarker for insulin resistance than leptin or adiponectin alone in adolescents? Children (Basel). (2022) 9:1193. doi: 10.3390/children9081193 36010082 PMC9406389

[25] Pedrosa C Oliveira BM Albuquerque I Simões-Pereira C Vaz-de-Almeida MD Correia F . Obesity and metabolic syndrome in 7–9 years-old Portuguese schoolchildren. Diabetol Metab Syndr. (2010) 2:40. doi: 10.1186/1758-5996-2-40 20537155 PMC2901245

[26] Levy-Marchal C Arslanian S Cutfield W Sinaiko A Druet C Marcovecchio ML . Insulin resistance in children: consensus, perspective, and future directions. J Clin Endocrinol Metab. (2010) 95:5189–98. doi: 10.1210/jc.2010-1047 20829185 PMC3206517

[27] Tagi VM Samvelyan S Chiarelli F . An update of the consensus statement on insulin resistance in children 2010. Front Endocrinol (Lausanne). (2022) 13:1061524. doi: 10.3389/fendo.2022.1061524 36465645 PMC9709113

[28] Constantinopoulos P Michalaki M Kottorou A Habeos I Psyrogiannis A Kalfarentzos F . Cortisol in tissue and systemic level as a contributing factor to the development of metabolic syndrome in severely obese patients. Eur J Endocrinol. (2015) 172:69–78. doi: 10.1530/EJE-14-0626 25336506

[29] Stimson RH Andersson J Andrew R Redhead DN Karpe F Hayes PC . Cortisol release from adipose tissue by 11β-hydroxysteroid dehydrogenase type 1 in humans. Diabetes. (2009) 58:46–53. doi: 10.2337/db08-0969 18852329 PMC2606892

[30] Gathercole LL Lavery GG Morgan SA Cooper MS Sinclair AJ Tomlinson JW . 11β-Hydroxysteroid dehydrogenase 1: translational and therapeutic aspects. Endocr Rev. (2013) 34:525–55. doi: 10.1210/er.2012-1050 23612224

[31] Sofer Y Osher E Abu Ahmad W Greenman Y Moshe Y Shaklai S . Cortisol secretion in obesity revisited: lower basal serum and salivary cortisol with diminished cortisol response to the low-dose ACTH challenge. Obes Facts. (2025) 18:217–25. doi: 10.1159/000543264 39778537 PMC12017752

[32] Mora M Adam V Palomera E Blesa S Díaz G Buquet X . Ghrelin gene variants influence on metabolic syndrome components in aged Spanish population. PloS One. (2015) 10:e0136931. doi: 10.1371/journal.pone.0136931 26375586 PMC4573319

[33] Müller TD Nogueiras R Andermann ML Andrews ZB Anker SD Argente J . Ghrelin. Mol Metab. (2015) 4:437–60. doi: 10.1016/j.molmet.2015.03.005 26042199 PMC4443295

[34] Wang YM Wu QX Zhou Q Chen Y Lei X Chen Y . Circulating acyl and des-acyl ghrelin levels in obese adults: a systematic review and meta-analysis. Sci Rep. (2022) 12:2679. doi: 10.1038/s41598-022-06636-3 35177705 PMC8854418

[35] Gupta AK Brashear MM Johnson WD . Prediabetes and prehypertension in healthy adults are associated with low vitamin D levels. Diabetes Care. (2011) 34:658–60. doi: 10.2337/dc10-1829 21282345 PMC3041202

[36] Tosunbayraktar G Bas M Kut A Buyukkaragoz AH . Low serum 25(OH)D levels are associated to higher BMI and metabolic syndrome parameters in adult subjects in Turkey. Afr Health Sci. (2015) 15:1161–9. doi: 10.4314/ahs.v15i4.15 26958017 PMC4765420

[37] Chen Z Qiu X Wang Q Wu J Li M Niu W . Serum vitamin D and obesity among US adolescents, NHANES 2011–2018. Front Pediatr. (2024) 12:1334139. doi: 10.3389/fped.2024.1334139 38836246 PMC11148364

[38] Barnard SA Pieters M De Lange Z . The contribution of different adipose tissue depots to plasma plasminogen activator inhibitor-1 (PAI-1) levels. Blood Rev. (2016) 30:421–9. doi: 10.1016/j.blre.2016.05.002 27233154

