# Supplementary material for: Adipokine, endocrine, and fibrinolysis-related alterations across abdominal obesity and metabolic syndrome phenotypes in young adults
Source: Front Endocrinol (Lausanne). 2026 Jul 28;17:1900593. doi: 10.3389/fendo.2026.1900593 (PMC13457479; doi:10.3389/fendo.2026.1900593)
Supplement: Supplementary file 1 [file Table1.docx]

Supplementary Material

# 1 Supplementary Tables

**Supplementary Table S1.** Bonferroni-adjusted post hoc pairwise comparisons for endocrine, adipokine, insulin-resistance, vitamin D, ghrelin, and PAI-1 biomarkers across metabolic phenotype groups.

| **Biomarker** | **Control vs AO** | **Control vs MetS** | **AO vs MetS** |
| --- | --- | --- | --- |
| Insulin | 0.011 | <0.001 | <0.001 |
| HOMA-IR | 0.069 | <0.001 | <0.001 |
| Adiponectin | <0.001 | <0.001 | 0.303 |
| Leptin | <0.001 | <0.001 | 0.123 |
| A/L ratio | <0.001 | <0.001 | 0.185 |
| Ghrelin | <0.001 | <0.001 | <0.001 |
| Cortisol | <0.001 | <0.001 | <0.001 |
| Vitamin D | <0.001 | 0.002 | 0.123 |
| PAI-1 | <0.001 | <0.001 | 0.018 |

Data are presented as Bonferroni-adjusted p-values from post hoc pairwise comparisons following significant Kruskal–Wallis tests. AO, abdominal obesity; MetS, metabolic syndrome; HOMA-IR, homeostasis model assessment of insulin resistance; PAI-1, plasminogen activator inhibitor-1; A/L ratio, adiponectin/leptin ratio.

**Supplementary Table S2**. Jonckheere–Terpstra trend tests across ordered phenotypes: control → AO → MetS.

| **Biomarker** | **Trend** | **Z (JT)** | **p-trend** | **r (= Z/√N)** |
| --- | --- | --- | --- | --- |
| Insulin | ↑ | 7.075 | <0.001 | 0.535 |
| HOMA-IR | ↑ | 6.749 | <0.001 | 0.510 |
| Cortisol | ↑ | 12.424 | <0.001 | 0.939 |
| Vitamin D | ↓ | −6.034 | <0.001 | −0.456 |
| Leptin | ↑ | 11.270 | <0.001 | 0.852 |
| Ghrelin | ↓ | −12.420 | <0.001 | −0.939 |
| PAI-1 | ↑ | 12.534 | <0.001 | 0.948 |
| Adiponectin | ↓ | −11.843 | <0.001 | −0.895 |
| A/L ratio | ↓ | −11.983 | <0.001 | −0.906 |

Data were analyzed using Jonckheere–Terpstra tests to evaluate ordered trends across the metabolic gradient from controls to abdominal obesity (AO) and metabolic syndrome (MetS). The p-trend value represents the two-sided p-value for the ordered trend. Effect size was calculated as r = Z/√N. Trend symbols indicate the direction of change across the ordered groups: ↑ increasing; ↓ decreasing. AO, abdominal obesity; MetS, metabolic syndrome.

**Supplementary Table S3.** Rank-based adjusted sensitivity analysis and Benjamini–Hochberg FDR correction.

| **Biomarker** | **Adjusted p for Group** | **q (BH-FDR)** |
| --- | --- | --- |
| Insulin | <0.001 | <0.001 |
| HOMA-IR | <0.001 | <0.001 |
| Cortisol | <0.001 | <0.001 |
| Vitamin D | <0.001 | <0.001 |
| Leptin | <0.001 | <0.001 |
| Ghrelin | <0.001 | <0.001 |
| PAI-1 | <0.001 | <0.001 |
| Adiponectin | <0.001 | <0.001 |
| A/L ratio | <0.001 | <0.001 |

Adjusted p-values for the overall group effect were obtained from rank-based general linear models using UNIANOVA on rank-transformed biomarkers. Study group was entered as a fixed factor, while sex, smoking status, and physical activity were included as adjustment variables. q-values represent Benjamini–Hochberg false discovery rate-adjusted p-values.

**Supplementary Table S4.** Female-only sensitivity analysis of endocrine, adipokine, insulin-resistance, vitamin D, ghrelin, and PAI-1 biomarkers across metabolic phenotype groups.

| **Variable** | **Female controls**  n = 74 median (IQR) | **Female AO**  n = 33 median (IQR) | **Female MetS**  n = 15 median (IQR) | **Kruskal–Wallis p** | **BH-FDR q** | **ε²** | **Female-only post hoc pattern** |
| --- | --- | --- | --- | --- | --- | --- | --- |
| Insulin (µIU/mL) | 7.25 (6.50–8.68) | 8.60 (6.90–12.30) | 15.60 (12.85–17.95) | <0.001 | <0.001 | 0.218 | C < AO < MetS |
| HOMA-IR | 1.35 (1.10–1.68) | 1.60 (1.20–2.30) | 3.00 (2.20–3.20) | <0.001 | <0.001 | 0.195 | MetS > C, AO |
| Adiponectin (ng/mL) | 301.60 (274.25–324.88) | 130.30 (103.40–195.20) | 108.60 (85.05–145.45) | <0.001 | <0.001 | 0.716 | C > AO, MetS |
| Leptin (pg/mL) | 701.50 (658.25–755.25) | 2104.00 (1900.00–2278.00) | 2356.00 (2201.00–2557.50) | <0.001 | <0.001 | 0.722 | C < AO, MetS |
| A/L ratio | 0.4336 (0.3785–0.4963) | 0.0718 (0.0523–0.0831) | 0.0512 (0.0337–0.0637) | <0.001 | <0.001 | 0.722 | C > AO, MetS |
| Ghrelin (pg/mL) | 182.45 (177.40–187.80) | 138.80 (136.10–140.60) | 116.60 (112.35–119.65) | <0.001 | <0.001 | 0.721 | C > AO, MetS |
| Cortisol (µg/dL) | 10.90 (10.43–12.30) | 16.30 (15.70–17.20) | 19.80 (19.30–20.70) | <0.001 | <0.001 | 0.724 | C < AO, MetS |
| Vitamin D (ng/mL) | 23.50 (21.33–24.80) | 16.40 (14.60–19.80) | 18.60 (13.65–21.90) | <0.001 | <0.001 | 0.367 | C > AO, MetS |
| PAI-1 (ng/mL) | 10.90 (9.90–12.10) | 20.40 (17.80–21.70) | 28.60 (26.60–29.80) | <0.001 | <0.001 | 0.749 | C < AO, MetS |

Data are presented as median (IQR). Between-group differences among female participants only were assessed using the Kruskal–Wallis test. The Benjamini–Hochberg false discovery rate procedure was applied across the nine female-only biomarker comparisons. Effect size is reported as epsilon-squared (ε²). Post hoc patterns are based on Bonferroni-adjusted pairwise comparisons following significant Kruskal–Wallis tests; only statistically significant pairwise differences are shown. This analysis was performed as a supportive sensitivity analysis because the AO group included only four male participants. C, control; AO, abdominal obesity; MetS, metabolic syndrome; HOMA-IR, homeostasis model assessment of insulin resistance; PAI-1, plasminogen activator inhibitor-1; A/L ratio, adiponectin/leptin ratio.

**Supplementary Table S5.** Adjusted single-biomarker logistic regression models for abdominal obesity and metabolic syndrome versus controls.

| **Comparison** | **Biomarker** | **Adjusted OR**  (95% CI) per 1-SD increase | **p value** |
| --- | --- | --- | --- |
| Abdominal obesity vs controls (n = 143) | PAI-1 | 1.67 × 10^4^  (131 to 2.13 × 10^6^) | **<0.001** |
|  | A/L ratio | 0.00433  (4.08 × 10^-4^ to 0.0459) | **<0.001** |
|  | Ghrelin | 0.00894  (0.00181 to 0.0441) | **<0.001** |
|  | Cortisol | 501  (49.7 to 5.05 × 10^3^) | **<0.001** |
| Metabolic syndrome vs controls (n = 138) | PAI-1 | 37.3  (8.61 to 162) | **<0.001** |
|  | A/L ratio | 0.00959  (0.00158 to 0.0581) | **<0.001** |
|  | Ghrelin | 0.0247  (0.0053 to 0.115) | **<0.001** |
|  | Cortisol | 49.0  (9.15 to 263) | **<0.001** |

Data are presented as odds ratios (ORs) with 95% confidence intervals (CIs) per 1-SD increase in biomarker concentration. Models were adjusted for sex, physical activity, and smoking status. Penalized logistic regression was applied because several biomarkers showed near-complete separation between groups. A/L ratio, adiponectin/leptin ratio; AO, abdominal obesity; CI, confidence interval; MetS, metabolic syndrome; OR, odds ratio; PAI-1, plasminogen activator inhibitor-1.
